# Supplementary material for: PD-L1 upregulation by IFN-α/γ-mediated Stat1 suppresses anti-HBV T cell response
Source: PLoS One. 2020 Jul 6;15(7):e0228302. doi: 10.1371/journal.pone.0228302 (PMC7337294; doi:10.1371/journal.pone.0228302)

Fig 1C. BALB/c or BALB/c HBV transgenic mice were treated with PBS, IFN-α (5 × 10^4 U/kg), IFN–γ (1.6 × 10^4 U/kg) every 3 days for 5 times, IHC analysis was performed for detection of PD-L1 expression levels in mouse livers. Scale bars, 50 μm.

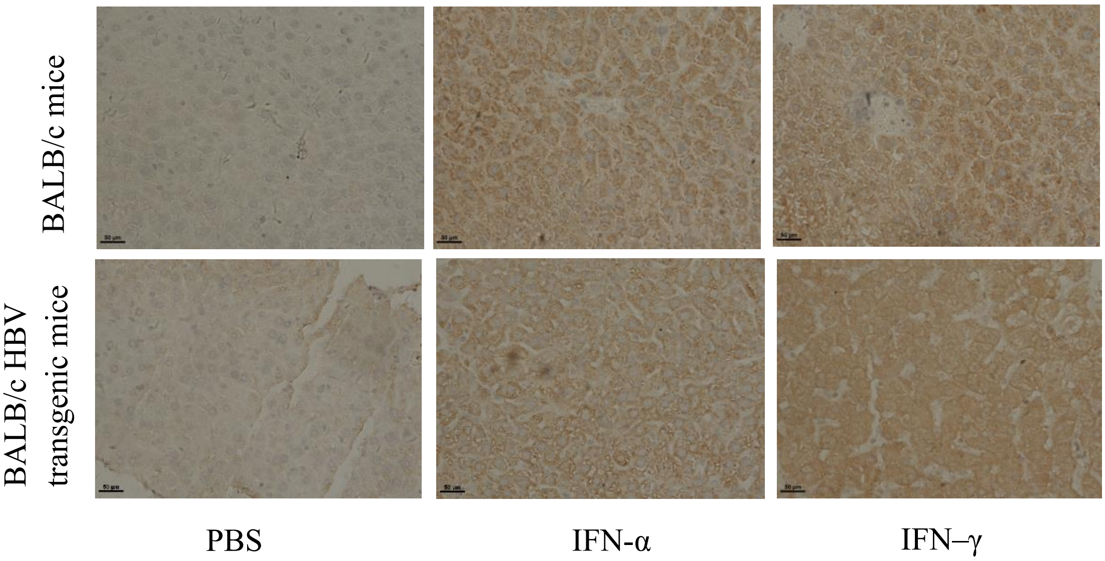

Supplement: S1 Fig — (PDF) [file pone.0228302.s001.pdf]
