# Supplementary material for: PD-L1 upregulation by IFN-α/γ-mediated Stat1 suppresses anti-HBV T cell response
Source: PLoS One. 2020 Jul 6;15(7):e0228302. doi: 10.1371/journal.pone.0228302 (PMC7337294; doi:10.1371/journal.pone.0228302)

Fig 2D: L02 cells treated with 80 U/ml IFN-γ or 50 U/ml IFN-α or PBS as control for 48h, Stat1 and phosphorylated Stat1 (p-Stat1) levels were determined by western blot.

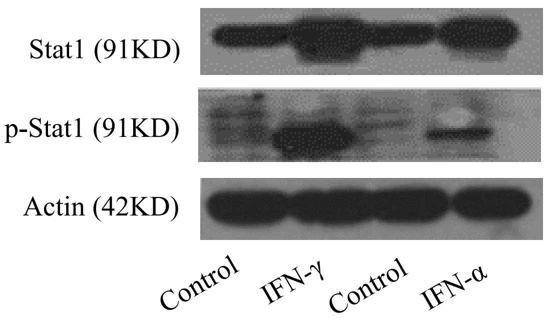

Supplement: S2 Fig — (PDF) [file pone.0228302.s002.pdf]
