# Supplementary material for: PD-L1 upregulation by IFN-α/γ-mediated Stat1 suppresses anti-HBV T cell response
Source: PLoS One. 2020 Jul 6;15(7):e0228302. doi: 10.1371/journal.pone.0228302 (PMC7337294; doi:10.1371/journal.pone.0228302)

Fig 4C. L02 cells treated with 80 U/ml IFN-γ or 50 U/ml IFN-α with or without (control) fludarabine for 48h, Stat1 and phosphorylated Stat1 (p-Stat1) levels were determined by western blot.

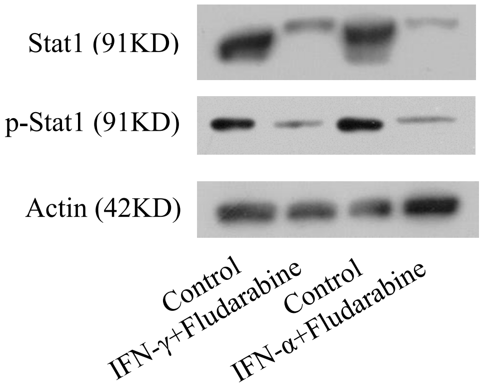

Fig 4D. L02 cells co-treated with 80 U/ml IFN-α or 50 U/ml IFN-γ, and Stat1 siRNA or control siRNA for 48 h. The mRNA and protein levels of PD-L1 were analyzed using real-time PCR and western blotting.

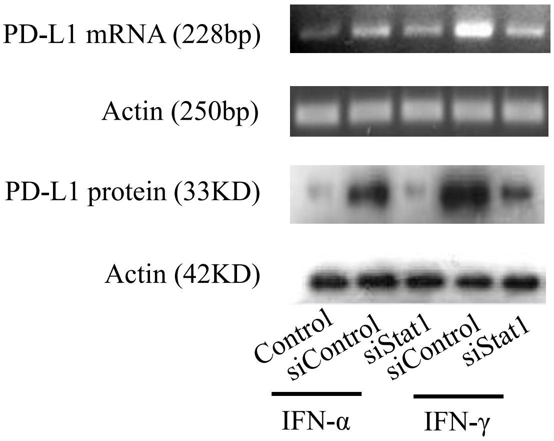

Fig 4E. BALB/c mice were treated with PBS, IFN-α (5×10^4 U/kg), and IFN–γ (1.6×10^4 U/kg), and fludarabine(40 mg/kg) every 3 days for 5 times. IHC analysis was performed for detection of PD-L1 expression levels in mouse livers. Scale bars, 50 μm.

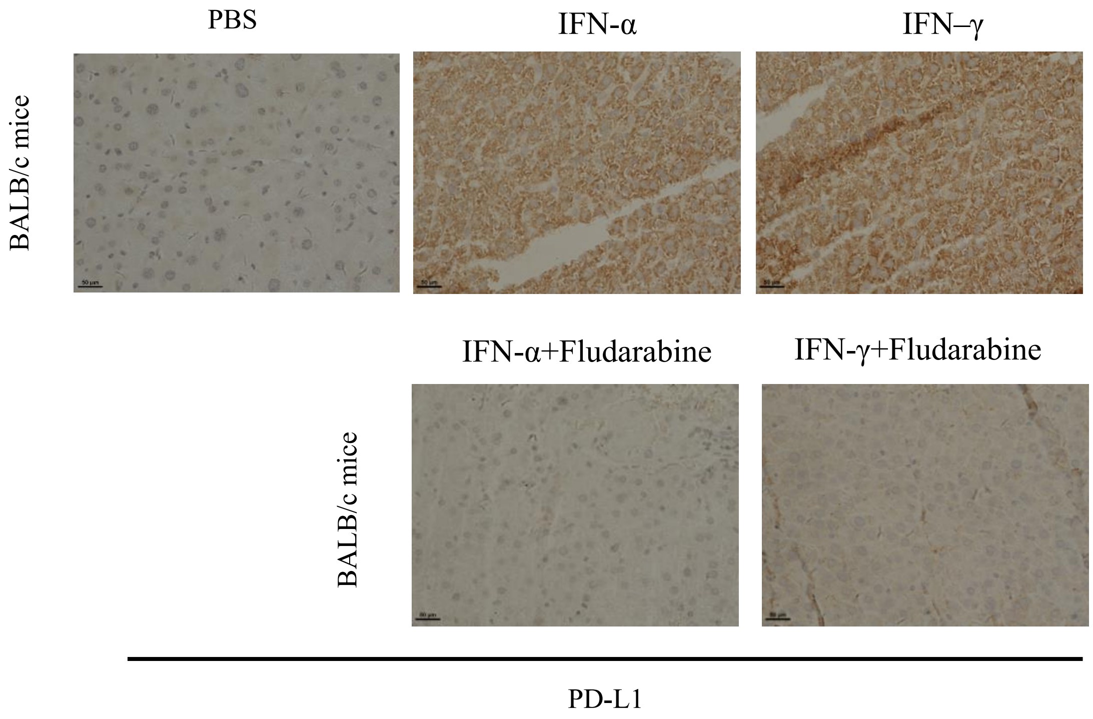

Supplement: S3 Fig — (PDF) [file pone.0228302.s003.pdf]
